# Genetics of the ceramide/sphingosine-1-phosphate rheostat in blood pressure regulation and hypertension

**DOI:** 10.1186/1471-2156-12-44

**Published:** 2011-05-13

**Authors:** Mogens Fenger, Allan Linneberg, Torben, Jørgensen, Sten Madsbad, Karen Søbye, Jesper Eugen-Olsen, Jørgen Jeppesen

**Affiliations:** 1 Copenhagen University Hospital at Hvidovre, Department of Clinical Biochemistry, Genetics, and Molecular Biology, Kettegaard All 26, 2650 Hvidovre, Denmark; 2 Research Centre for Prevention and Health, Copenhagen University Hospital at Glostrup, Ndr. Ringvej 57, 2600 Glostrup, Denmark; 3 Copenhagen University Hospital at Hvidovre, Department of Endocrinology, Kettegaard All 26, 2650 Hvidovre, Denmark; 4 Copenhagen University Hospital at Hvidovre, Clinical Research Unit, Kettegaard All 26, 2650 Hvidovre, Denmark; 5 Copenhagen University Hospital at Glostrup, Deparement of Caridology Ndr. Ringvej 57, 2600 Glostrup, Denmark

## Abstract

**Background:**

Several attempts to decipher the genetics of hypertension of unknown causes have been made including large-scale genome-wide association analysis (GWA), but only a few genes have been identified. Unsolved heterogeneity of the regulation of blood pressure and the shortcomings of the prevailing monogenic approach to capture genetic effects in a polygenic condition are the main reasons for the modest results. The level of the blood pressure is the consequence of the genotypic state of the presumably vast network of genes involved in regulating the vascular tonus and hence the blood pressure. Recently it has been suggested that components of the sphingolipid metabolism pathways may be of importance in vascular physiology. The basic metabolic network of sphingolipids has been established, but the influence of genetic variations on the blood pressure is not known. In the approach presented here the impact of genetic variations in the sphingolipid metabolism is elucidated by a two-step procedure. First, the physiological heterogeneity of the blood pressure is resolved by a latent class/structural equation modelling to obtain homogenous subpopulations. Second, the genetic effects of the sphingolipid metabolism with focus on *de novo *synthesis of ceramide are analysed. The model does not assume a particular genetic model, but assumes that genes operate in networks.

**Results:**

The stratification of the study population revealed that (at least) 14 distinct subpopulations are present with different propensity to develop hypertension. Main effects of genes in the *de novo *synthesis of ceramides were rare (0.14% of all possible). However, epistasis was highly significant and prevalent amounting to approximately 70% of all possible two-gene interactions. The phenotypic variance explained by the ceramide synthesis network were substantial in 4 of the subpopulations amounting to more than 50% in the subpopulation in which all subjects were hypertensive. Construction of the network using the epistatic values revealed that only 17% of the interactions detected were in the direct metabolic pathway, the remaining jumping one or more intermediates.

**Conclusions:**

This study established the components of the ceramide/sphingosine-1-phosphate rheostat as central to blood pressure regulation. The results in addition confirm that epistasis is of paramount importance and is most conspicuous in the regulation of the rheostat network. Finally, it is shown that applying a simple case-control approach with single gene association analysis is bound to fail, short of identifying a few potential genes with small effects.

## Background

Hypertension, defined as office blood pressure (BP) measurements of 140/90 mm Hg or greater, affects 30% of the adult population, and is a major risk factor for stroke, heart disease, and end-stage renal disease [1]. Hypertension arises as a consequence of altered activity in signal transduction pathways and interactions of complex intra- and intercellular processes [2,3]. However, the exact mechanism and causes of hypertension are unknown in 95% of the cases (essential hypertension). In the remaining 5% of cases, the cause of hypertension is secondary to various conditions including endocrine disorders as well as drug-induced hypertension [4]. Although several clinical and biochemical variables are correlated to hypertension [5-7] the causes of essential hypertension remains elusive. It has been estimated that 30-60% of the blood pressure variation in humans is caused by genetic factors [8], but so far the genetic mechanisms underlying human essential hypertension have not been defined [9]. Several strategies including genome-wide association (GWA) studies [8,10,11] have been applied, but despite these huge efforts only a few potential genes have been associated with essential hypertension. In particular the large GWA studies have regrettably falling short of explaining the full genetic picture associated with hypertension [12-16]. The reasons for this are numerous, one of the most important being that most studies do not includes gene-gene interactions in the analysis (power issues being the main cause), despite the fact that essential hypertension is a polygenic trait influenced by an unknown number genes probably running in the hundreds. As in all other biological processes the regulation of the blood pressure is defined in networks of interacting genes, in which the activity and impact on the blood pressure depends on the genetic variety in various parts of the networks. This genetic variety is expressed as phenotypic heterogeneity and essential hypertension is merely a clinical end-point term for the diverse states of the genetic pathways of blood pressure regulation.

Several methods have been developed to account for population and genetic heterogeneity and epistasis [17]. The basic hypothesis is that any observable phenotype of a complex biological system by necessity is determined by the complexity of its underlying biochemical organisation and the signalling network operating within it. It is the entire network behaviour and not a single, specific variable that determines the physiological outcome. As all biochemical processes are governed by the genes encoding for the executing proteins, the interactions of the proteins reflect the genotype of the individual and the variations in the genotype define the range within which the executer operates. Thus, the entire genotype contributes to the biochemical and physiological outcome and to what extent an individual will actually develop a clinical significant state like diabetes [17] or hypertension. Here we apply the approach previously implemented in diabetes mellitus type 2 to explore the genetic network of the ceramide/sphingosine-1-phosphate (Cer/S1P) rheostat on several measures of blood pressure in a large randomly selected population partitioned into homogenous subpopulations by quantitative physiological variables in a latent class/structural equation model framework [17]. Sphingolipids influence the vascular tone, but the effects are controversial as both vasodilatory and vasoconstrictive effects have been reported for the essential metabolites in the rheostat network, ceramides and sphingosine-1-phosphate [18-21]. Thus, the role of the rheostat in hypertension is far from being resolved [22]. The analysis presented here is focused on the integrated *de novo *synthesis of ceramides and extended to construct the metabolic network of the rheostat. This is in contrast to the previous study on diabetes mellitus [17] in which the genes included did not represent a composite metabolic network, but was selected from association studies of diabetes mellitus type 2. The structure of the Cer/S1P-rheostat network was confirmed, but in addition it was revealed that the regulatory components of the network are far more conspicuous than the direct metabolic pathways.

## Methods

### Population

The study population is the Danish leg of the international MONICA-study [23,24]. In brief, randomly selected women and men from the County of Copenhagen were enrolled in 1982 and re-invited for a reexamination in 1993-94 including 2,656 subjects [25]. Some of the subjects only had sparse or no data on the variables used in this study and were excluded slightly reducing the study population to 2,523 individuals. Fasting levels of glucose, insulin, lipids, and apolipoproteins (ApoA1, ApoB and ApoE), C-reactive protein (CRP), B-type or brain natriuretic peptide (BNP), soluble urokinase activator receptor (Supar), aortic pulse wave velocity (compliance) and aortic stiffness (Aasi) [26] as well as anthropometric measures as waist-hip circumference (WH) and body mass index (BMI) were included in the latent profile analysis LPA-SEM modelling (see below). Insulin resistance (HOMAres) was calculated using fasting glucose and insulin levels [27]. Blood pressures were measured by a standard "office" procedure in sitting and supine position. In addition, a 24-hr ambulatory blood pressure monitoring was performed in a subgroup consisting of 1,829 participants as described in detail elsewhere [28]. At least 13 readings of systolic and diastolic blood pressure during the day and at least 6 readings of systolic and diastolic blood pressure during the night were obtained. Finally, ultrasound blood pressure evaluations were performed in both arms as previously described [26]. The variables of the study population are summarized in Table 1.

**Table 1:** Summary of the indicators and covariates in the Monica study included in the LCA-SEM analysis.

	Mean (SD)		
	All	Women	Men
Number	2,523	1,257	1,266
Age	43.6 (10.8)	43.2 (10.8)	44.0 (10.7)
*Blood pressure measurements (mm Hg):*			
			
Sitting systolic	129 (19)	127 (19)	132 (19)**
Sitting diastolic	82 (11)	81 (10)	84 (11)**
Supine systolic	129 (18)	127 (19)	131 (17)**
Supine diastolic	80 (10)	78 (10)	82 (10)**
Day systolic	131 (14)	127 (14)	134 (13)**
Day diastolic	78 (10)	75 (10)	81 (9)**
Night systolic	112 (14)	110 (15)	115 (14)**
Night diastolic	64 (9)	62 (9)	66 (9)**
24 hour systolic	126 (14)	123 (14)	129 (13)**
24 hour diastolic	74 (9)	71 (9)	77 (9)**
Ultrasound left site systolic	131 (21)	130 (22)	132 (19)**
Ultrasound left site diastolic	79 (11)	78 (12)	81 (11)**
Ultrasound right site systolic	127 (21)	126 (22)	128 (19)*
Ultrasound right site diastolic	78 (11)	77 (11)	80 (11)**
Ultrasound mean systolic	129 (20)	128 (21)	130 (18)*
Ultrasound mean diastolic	79 (11)	77 (11)	80 (10)**
Total cholesterol (mmol/L)	6.2 (1.1)	6.1 (1.1)	6.2 (1.1)
LDL cholesterol (mmol/L)	4.0 (1.0)	3.9 (1.0)	4.1 (1.0)**
VLDL cholesterol (mmol/L)	0.65 (0.37)	0.58 (0.29)	0.72 (0.42)**
T riglycerides (mmol/L)	1.46 (1.04)	1.29 (0.88)	1.63 (1.16)**
HDL cholesterol (mmol/L)	1.44 (0.41)	1.60 (0.43)	1.29 (0.34)**
WH ratio	0.88 (0.09)	0.82 (0.06)	0.94 (0.06)**
BMI (kg/height^2^)	26.0 (4.2)	25.4 (4.5)	26.5 (3.8)**
Insulin (pmol/L)	38 (33)	33.5 (26.4)	42 (38)**
Glucose (mol/L)	4.9 (1.1)	4.7 (0.9)	5.1 (1.2)**
HOMAres	1.46 (1.73)	1.24 (1.22)	1.69 (2.10)**
ApoA1 (mmol/L)	52.6 (21.4)	56.0 (22.6)	49.6 (20.0)**
ApoB (mmol/L)	0.73 (0.24)	0.70 (0.24)	0.77 (0.24)**
ApoE (mmol/L)	2.25 (1.18)	2.30 (1.23)	2.20 (1.13)*
Compliance	0.57 (0.15)	0.59 (0.15)	0.55 (0.15)**
Aasi	11.1 (3.3)	10.6 (3.3)	11.6 (3.3)**
Left ventricular mass	80.5 (21.1)	73.2 (17.5)	88.0 (21.7)**
Supar (mg/L	4.21(1.33)	4.39 (1.31)	4.01 (1.32)**
BNP (ng/L)	76.0 (135.1)	89.5 (115.7)	61.7 (152.2)**
CRP (mg/L)	3.2 (5.5)	3.4 (6.1)	3.1 (5.0)

Day, Night and 24 hour blood pressures are mean of several measurements dyring the time period.

*p < 0.01; **p < 0.001 (difference between gender).

The research was performed according to the Helsinki declaration. The study was approved by the Ethics Committee of the Greater County of Copenhagen, Denmark, implying that informed consent was given by all participants.

### Model assumptions

No assumptions are made for the distribution of traits in the basic, heterogeneous study population, but it is assumed that the study population consists of a mixture of more homogeneous subpopulations in which the physiological variables defining and characterizing the subpopulations are normal distributed. Variables may not be exactly normally distributed in a tissue or an organism because of asynchrony of the dynamic processes in the cells [29], but the maximum likelihood ratio estimation method used here is robust to minor deviations from normality (increased skewness and kurtosis) [30,31].

All subjects are assumed to possess the same basic genetic structures and networks, but vary in expression because of variability in the genome, including single nucleotide polymorphisms (SNPs), deletions, insertions, copy-number variation, non-coding RNA complexes, epigenetic diversity etc. No genetic structure or model is assumed *a priori*, but are embedded in the latent structural equation model (SEM) as the basic network and defines the kernel of the cellular processes. The genotype in individual subjects is fixed allocating an individual to a particular subpopulation of similar physiological expression. It is therefore implicit that no subject transitions between subpopulations are possible. Only transitions from one level to another level of the blood pressure would be possible within the subpopulation, depending on the load of non-genetic factors and within the limits defined by the subpopulation-specific genotype, i.e. the non-genetic factors operate within the limits of a genetic framework that physiologically cannot be exceeded.

### Resolving heterogeneity in the population

**Figure 1:**
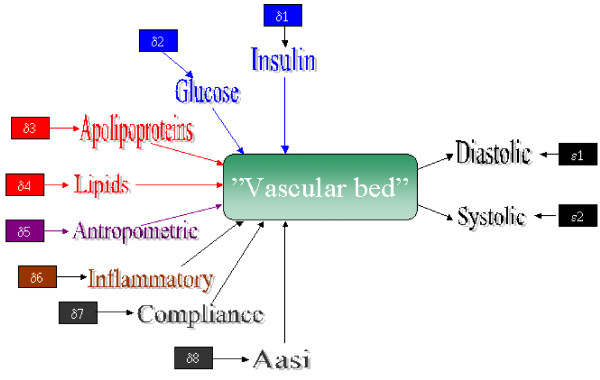
**The figure illustrates the simplified blood pressure model**. The centre of the model is the latent "Vascular bed", which is in quotation mark as it represent a latent structure harboring all kinds of metabolic, physiologic and genetic processes in the arterial vessels. The physiological state of the vascular bed govern the diastolic and systolic blood pressures, which are the indicators in the model. The physiological state of the arterial vessels are influenced by several covariates as indicated in the figure. The covariates Apolipoproteins, Lipids, Antropometric, and Inflammatory covers several items each, but are not shown separately (see the text for details). Insulin and glucose is the variables used to calculate insulin resistance (HOMAres), but is not shown here as the measure is partly an intrinsic parameter in the vascular cells as well as many other tissues.

The population was partitioned by an integrated latent class/structural equation modelling (LPA-SEM) as previously described [17,32,33]. In this study it is assumed that the population consists of a mixture of subpopulations within which all variables in the model are presumed to be normally distributed (although other distributions can be assumed) and only correlated through the latent SEM variable as previously described [17]. The model is schematically shown in Figure 1. Mathematically the model is formulated as *f*(**y**|**z**) = ∑_x_*π*(**x**|**z**)*f*(**y**|**x**, **z**) where we are modelling the probability density of observing the indicators **y **(diastolic and systolic blood pressures) given a set of covariates **z **(lipids, insulin etc.). A latent variable is interspersed between the indicators and covariates (**x**, "Vasular bed" in Figure 1) i.e. the indicators are not directly regressed on the covariates. The latent SEM variable is a composite variable as it includes a plethora of processes that are not measured directly. As such, all distinct processes defined in small integrated networks are latent, but at the moment we are only modelling a summary latent variable consisting of an ensemble of (often undefined) membrane composition, organelles, signalling and regulatory pathways, genetic structures and variations, and any biochemical processes that are involved in the viability and dynamic behaviour of the cell. In this formulation *π*(x|**z**) is the probability of having a certain set of values for the discrete latent variable (**x) **given an individual's observed covariates. The conditional *π*(x|**z**) is equivalent to the probability for an individual to belong to a subpopulation and sums to 1 over all subpopulations for each subject. At the population level, *π*(x|**z**) reflects the relative size of a subpopulation.

The outcome of this modelling is a classification of the population sample in mutually exclusive subpopulations with significantly distinct physiological states differing in their propensity to evolve into the clinical endpoint hypertension. However, the clinical endpoint *per se *was not entered in the model. Analysis were performed using Mplus v 5.1 [30] attempting to obtain normal distributions of the variables and using the adjusted Bayesian information criteria (BIC) as the goodness-of fit measure and the entropy as defined in the programme as a measure of accuracy of the partitions. BIC evaluates the likelihood of the model compared to the null-model and corrects for sample size. The model with the lowest BIC value is considered the model of choice. If two (or more models) are equivalent or only marginal different the entropy as a measure of the errors in the models provide guidance of which model to select (see Results for evaluation of the models).

### Genotyping

**Figure 2:**
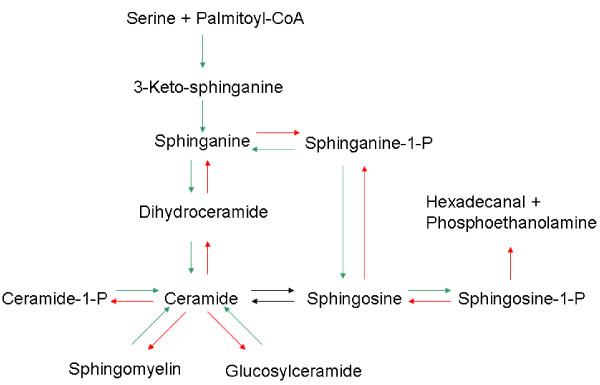
**Schematic outline of the ceramide/sphingosine-1-phosphate rheostat**. Single arrows indicate irreversible processes, i.e. no enzymatic activities have been detected that reverses the metabolic pathway depicted by the arrows. Of particular interest is the degradation of sphingosine-1-phosphate to hexadcanal and phophoethanolamine which is a sink for sphingosine-1-phosphate irreversibly terminating its signalling activity.

The entire study population was genotyped for single nucleotide polymorphisms (SNPs) related to the *de novo *synthesis of ceramides and the kernel of the ceramide/spingosine-1-phosphate rheostat. Many more genes are involved in the sphingolipid metabolism, many of which we have none or only limited knowledge about. Therefore the focus of this study was restricted to the well-described basic metabolism of ceramides and sphingosine-1-phosphate (see Figure 2). Missense or frameshift mutations in all exons in the selected genes were included in the genetic analysis. For exons in which no missense mutations are present synonymous mutations were genotyped to obtain maximum coverage of the genes. The details of the genes and SNPs are listed in Tables T1 and T2 (see Additional files 1 and 2). The genotyping was performed by KBioscience, Hoddesdon, UK. Genotyping was validated in 5% of the population for all genes and all genotypes were confirmed. Missing genotypes were in the range 0.07% to 3.4% (mean 0.88%, median 0.84%). No imputations of missing genotypes were attempted.

### Epistasis and network constructions

The rheostat network was re-constructed using variance decomposition of two-SNP interactions. The SNP-SNP/gene-gene interactions (epistasis) and heritabilities were calculated as described [17,34]. Briefly, the procedure described by Lynch and Walsh [34] is a simple step-wise decomposition of genetic values and effects used to calculate variances. Single SNP additive and dominance variance as well as two-SNP additive-additive, additive-dominant and dominant-dominant variance are calculated. The variance decomposition in these formulations was orthogonal [35,36], that is the estimates obtained are consistent and unbiased provided Hardy-Weinberg equilibrium holds. Tests for significance is described in [17] and references therein where corrections of flaws in the original procedures have been corrected.

All algorithms were encoded in R and run in a Python shell in Linux (Ubuntu v. 9.4).

The LPA-SEM partition using blood pressure measurements in the supine position as indicators was used in network construction as this partition generated the best fitting model (see Results). Significant genetic variances of the supine diastolic and systolic blood pressure measurements were used to select subsets of two-SNP/gene interactions in the construction of the network, i.e. the fraction of epistatic variances relative to total phenotypic variance represented the links and the proteins (enzymes) constituted the nodes in the network. Centrality indices (closeness, betweenness, centroid, stress, Katz and vitality) were calculated as summarized in Junker et al [37]. Calculations and network constructions were performed using the CentriBiN [37] and Pajek [38] softwares. Linkage disequilibrium and haplotype inference were performed using the Arlequin software [39].

### Statistics

All basic statistics were performed in SPSS v18.0. Bonferoni correction for multiple testing with a nominal p-value of 0.05 was applied to all analysis. In the final model with 14 subpopulations the cut-off p-value is 2*10^-6^. This value may differ slightly in the other models depending on the number of subpopulations defined. The Linux R-front-end RKWard http://rkward.sourceforge.net was implemented in tests for normality of the variables [40].

## Results

The distribution of the diastolic and systolic blood pressures and all the co-variates entering the partition models except Aasi were significantly non-normal in the overall population (see Additional file 3). Using the Shapiro-Wilk test for normality of Aasi suggested that this variable is non-normally distributed with a p-value of 4.4*10^-3^, but was normal distributed when other tests for normality were used (Lilliefors/Kolmogorov-Smirnov and Anderson-Darling [40]). Thus, the study population is extremely heterogeneous suggesting that the population is composed of a mixture of physiological different subpopulations.

### Resolving heterogeneity in the population

The covariates significantly influencing the LPA-SEM classification includes measures for insulin-glucose metabolism (HomaRes, waist-hip ratio), lipid metabolism (VLDL, apolipoprotein A1, B, and E), markers for low-grade inflammatory disease (C-reactive protein (CRP), soluble urokinase activator receptor (Supar), brain natriuretic peptide (BNP)), and measures of vessel stiffness (compliance, Aasi). Most of these entered as covariates in the seven models in the LPA-SEM partitions, but may vary in rank (Table 2). VLDL was included in all models and is particularly interesting as it and other lipoproteins are composite particle harbouring ceramides and sphingosine-1-phosphate [41,42]. The entropy measures indicated a rather high, although not complete, accuracy of the partitions.

**Table 2:** Summary of the LCA-SEM partition using systolic and diastolic blood pressure as indicators

			Entropy^b^				
Model	Number of subpopulations	Co-variates in decreasing order of significans^a^	With covariates	Change	% Normalized^d^		Correlation of indicators^e^
				Excluding CRP and BNP			
*Average 24 hours*	14	CRP HomaRes Compliance Supar VLDL Aasi WH BNP ApoE	0.789	-2.3%	66.9%	81.8%	71.4%
*Average daytime*	12	CRP HomaRes Compliance Supar VLDL WH BNP ApoE Aasi ApoA1	0.788	+5.3%	61.1%	75.0%	83.3%
*Average night*	13	CRP HomaRes Compliance Supar VLDL BNP ApoE	0.747	-9.3%	64.1%	79.5%	84.6%
*Sitting (office BP)*	11	CRP Compliance VLDL Homares Age BNP BMI ApoA1	0.724	-1.0%	50.0%	69.1%	81.8%
*Supine*	14	HomaRes BNP Compliance Supar VLDL CRP Age BMI ApoA1	0.792	+2.5%	68.8%	80.5%	100.0%
*UL4^c^*	12	HomaRes CRP Supar BNP VLDL ApoA1 ApoE	0.759	-5.8%	47.0%	65.2%	80.6%
*UL2^c^*	14	HomaRes BNP Compliance Supar VLDL CRP ApoA1 Age BMI	0.801	+5.8%	68.7%	80.8%	28.6%

^a ^CRP, C-reactive protein; HomaRes, insulin resistance;VLDL, very low densitity lipoprotein; Aasi, vessel stifness; WH, waist-hip ratio; BNP, B-type or brain natriuretic peptide; BMI, body mass index; Apo, apolipoprotein; Supar, soluble urokinase activator receptor.

^b ^Entropy for the full model as defined in the MPlus programme (1 - standard entropy) for the error structure. Changes signifies deviation from the model without covariates.

^c ^Ultrasound recording of systolic and diatolic blood pressure: UL4, left and rigth measurements are seperate indicators;

UL2, average of left and right systelic and diastolic blood pressure, respectively, are used as indicators

^d ^Fraction of traits in all classes normalized by the LCA-SEM partition. Only the traits listed and the mode of blood pressure measurements are included in the calculation

^e ^Fraction of indicators not correalted after LCA-SEM partition

A basic assumption in the modelling is that the population is composed of a mixture of normally distributed subpopulations. The purpose of the LPA analysis was to disentangle the physiological heterogeneity of the population and to define more homogeneous subpopulations (i.e. defining more precise phenotypes) and this was obtained to a large extent (Table 2). Disregarding the inflammatory markers BNP and CRP, which are rather non-specific variables and had extremely high variance or standard errors (Table 1), the fraction of normalized traits increased up to 80%. Most notably, the indicators in the models (the blood pressure measurements) were all normalized without exception. This was not the case for the models without covariates, where 4 of the 7 models did not normalize the indicators. Comparison of the models with and without covariates included revealed a small, but significant influence on the error structure of the co-variates indicated by the modest changes of the entropy measure (Table 2). However, inclusion of the co-variates reduced the adjusted BIC values approximately 25% (not shown). Thus, the indicators imposed the most weight on the basic partition, while the covariates improved the goodness-of-fit of the model, and normalized the indicators fulfilling a major assumption in the LPA-SEM model. The distributions of the covariates were generally "uniform" and did not have any predictive value to the assignment of subjects to the subpopulations. The analysis of models without the co-variates i.e. a pure finite mixture model [33] revealed a significantly different distribution of the subjects between the subpopulations than the models including the covariates (p < 0.001). This is shown for t blood pressure measured in supine position (the Supine model) in Table 3. The distribution was more "uniform" for the models including covariates and in all models the highest and lowest numbers of subjects in the subpopulations were assigned in the pure finite mixture models.

**Table 3:** Number of significant interactions in the subpopulations in the Supine model

Partition:	model with covariates					Supine model without covariates					
**Trait:**		**Systolic**		**Diastolic**				**Systolic**		**Diastolic**	

**Subpopulations**	**No cases**	**Interactions**	**Fraction**	**Interactions**	**Fraction**		**No cases**	**Interactions**	**Fraction**	**Interactions**	**Fraction**

1	39	307	32,49%	307	32,49%		27	331	35,03%	306	32,38%

2	111	318	33,65%	316	33,44%		113	325	34,39%	325	34,39%

3	259	298	31,53%	294	31,11%		625	323	34,18%	335	35,45%

4	33	230	24,34%	334	35,34%		104	338	35,77%	338	35,77%

5	189	257	27,20%	257	27,20%		1	0	0,00%	0	0,00%

6	301	321	33,97%	310	32,80%		30	320	33,86%	320	33,86%

7	132	306	32,38%	307	32,49%		23	262	27,72%	332	35,13%

8	89	238	25,19%	238	25,19%		89	304	32,17%	305	32,28%

9	237	275	29,10%	276	29,21%		332	231	24,44%	238	25,19%

10	141	334	35,34%	337	35,66%		279	295	31,22%	295	31,22%

11	163	295	31,22%	302	31,96%		7	0	0,00%	0	0,00%

12	40	336	35,56%	336	35,56%		544	226	23,92%	232	24,55%

13	166	307	32,49%	306	32,38%		73	285	30,16%	285	30,16% 32,28%

14	21	174	18,41%	148	15,66%		19	159	16,83%	305	

											

Sum	1.921	3.996		4.068			2.266	3.399	3.616		

Average		285,43	30,20%	290,57	30,75%			242,79	25,69%	258,29	27,33%

Variance		2.115		2.498				13.074		13.012	

Average Total		30,20%		30,75%				25,69%		27,33%	

Minimum	21	174	18,41%	148	15,66%		1	0	0,00%	0	0,00%

Maximum	301	336	35,56%	337	35,66%		625	338	35,77%	338	35,77%

											

Systolic versus diatolic blood pressure^b^				Number of interactions					Number of interactions		

			p-value	0,78					0,72		

											

Supine model with and without covariates^c^											

			Systolic	Diastolic							

F-test		p-value	0,0024	0,0055							

											

Number of total interactions detected					Full model			Model without co-variates			

					Interactions	Fraction		Interactions	Fraction		

				Systolic	676	68,28%		695	70,20%		

				Diastolic	681	68,79%		695	70,20%		

a The fraction is calculated as the number of significant interactions detected realtive to the maximal number of interaction possible

b Test of difference in number of interactions between diastolic and systolic blood pressures

c Test of the number of significant interactions in the two models. The number of interactions in the model with covariates (the fulle model)

is significant higher than in the resticted model without covariates.

The indicators were strongly correlated for all the different measures of blood pressure (p < 0.001). The modelling approach tries to explain the correlation between the indicators by a common latent variable, that is, correlation between observed variables should vanish in a perfect classification. Indeed, the correlations between the indicators (diastolic and systolic blood pressure) in the subpopulations in each model were non-significant to a large extent except for the UL2 model (Table 2). Most of the classes with correlated indicators are large suggesting that heterogeneity has not been resolved in these classes. In a few classes with correlated indicators the number of subjects was in the low end suggesting either that the models were overfitted or that (unknown) covariates of importance were not included. Only the Supine model provided non-correlation between the indicators in all the classes. In this model without including the covariates the diastolic and systolic blood pressures were correlated in 5 of the 14 subpopulations indicating that modelling pure finite mixture models was insufficient and produced incorrect results.

The systolic and diastolic blood pressure differed significantly between the subpopulation in each model and reached 100% in most of the models the exceptions being the Night and UL2 models (se Additional file 4).

Taken together the above results suggest that the Supine model provides the most accurate description of the population and this model will be the benchmark model in the following. It should remembered that decisions about which model is the "best" in LPA.-SEM procedures may be subtle. It could be argued that some of the other models may be "better" or at least "as good". The Supine model is, however, one of the best models fulfilling most criteria and assumptions in the LPA-SEM approach in particular a high entropy and non-correlations of the indicators. A practical argument for focusing on the Supine model is that supine blood pressure measurements are readily available, which make the further analysis of the Supine model more appropriate to future studies.

**Figure 3:**
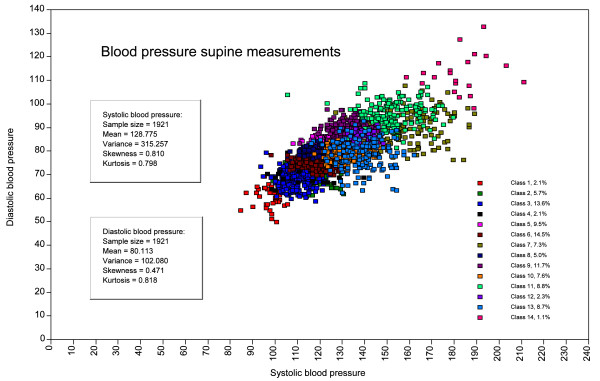
**Partition of the population into 14 subpopulations using the supine blood pressure measurements as indicators and including the covariates in LPA-SEM procedure**. The figure shows the distribution of the 14 subpopulations and the overall statistics of the study population. Similar distributions were obtained in the other models. A view of the subpopulations in the Supine model can be seen in Additional file 5.

The distribution of subjects in the 14 subpopulations in the Supine model is shown in Figure 3. Similar patterns were obtained for all the other models in Table 2 (not shown). The distribution of the subjects in the subpopulations can be viewed in Figure F1 (see Additional file 5) together with basic statistical measures. Assignment of subjects to subpopulations differed significantly between the models suggesting that the mode of blood pressure measurements reflects differences in physiology (e.g. sitting versus supine) and/or differences in techniques (e.g. sphygmanometer versus ultrasound).

#### Genetic analysis

The focus in this study was the *de novo *synthesis pathway of ceramides, and comprehensive coverage was attempted for this part of the rheostat network. The selected genes and single nucleotide polymorphisms (SNPs) included are listed in Tables T1 and T2 (see Additional files 1 and 2). The SNPs were extracted from dbSNP database (NCBI) as of March 2009. Genotyping of five of the 106 SNPs was unsuccessful despite several attempts to genotype them. In addition, 56 of the SNPs turned out to be monomorphic and thus were non-informative. The remaining 45 SNPs were informative and included in the genetic analyses. The number of possible two-SNP interactions is thus 990, which are evaluated in all subpopulations for all the modes of blood pressure measurements.

Most of the genes were represented by more than one SNP. Several of the SNPs in the same gene were in linkage disequilibrium (LD) (se Additional file 2). However, 50% of the possible intragenic linkage disequilibria were not detected. The intergenic LDs amounted to 4.11% of all possible, despite that all the genes are located on different chromosomes. In the Supine subpopulations (which are the show-case of this presentation) the intragenic LDs were in the range of 2.5-42.5%, while the intergenic LDs were reduced in the most of the subpopulations (range 0 - 4.21%). All SNPs except rs41303970 and rs7302981 were in Hardy-Weinberg equilibrium in the study population. This may be due to technical difficulties, but as the genotyping was of excellent quality (see Methods) it is not a likely explanation. Rather, the two exceptions may reflect that the study population consisted of a mixture of subpopulations in which the SNPs may or may not be in Hardy-Weinberg equilibrium. Therefore, all SNPs are included in the analysis of all the subpopulations, but any SNP not in equilibrium were excluded from variance decomposition analysis. This will be the case for all SNPs not just the two SNPs not in equilibrium in the general study population, which may in fact be in equilibrium in the subpopulations.

Intragenic interactions could be caused by physical linkage (LD) or because the partition by the LPA-SEM procedure group variants defining the physiological state (blood pressure). The interaction analysis cannot discern between these two possibilities and therefore the intragenic SNPs were disregarded in the further analysis of epistasis or represented by the SNP with the largest interactions with other genes in the centrality and network analysis (see below).

### Gender partition

Although gender represents a rather crude partition of a study population it served the purpose of providing an overall view of the genetic structure of the Cer/S1P rheostat related to blood pressure. Two-locus interactions were evaluated for all modes of diastolic and systolic blood pressure measurements. Approximately one third of the possible number of interactions (990 for each mode of blood pressure measurements and each gender) were significant in men and slightly more in women (see Additional file 6). The SNP-combinations differed among the models summing the total number of significant epistasis to slightly above half the possible number of interactions. Except for 11 interactions all the common interactions in systolic and diastolic blood pressure were the same (not shown). None of the genes showed any main effects (adjusted p-value <5.1e-05), but all the genes participated significantly in various and numerous interactions, although the phenotypic variances accounted for were very small for each two-gene interactions (average 0.001 and less than 0.012).

### LPA-SEM partitions

The LPA-SEM partition significantly reduced the variance of the blood pressures as shown for the Supine model in Table T6 (Additional file 7). The number of possible epistatic interactions were 990 for the 45 SNPs included, but the actual number of interactions were less and varied between models and subpopulations as the partitions left SNPs monomorphic in some of the subpopulations and because in some cases the traits (blood pressures or covariates) were missing (see Additional files 8 and 9). In subpopulations with less than 30 subjects only two subpopulations failed to detect any substantial interaction: in the subpopulation with 6 subjects (the Night model, subpopulation 1, not shown) no interactions were detected; and in the subpopulation with 22 subjects (the UL2 model subpopulation 2, not shown), only one interaction where detected (between rs455225532 in CerS1 and rs17160349 in CerS4). The latter could be interpreted as a false positive result, but the genetic variance accounted for was 14.3% of the phenotypic variance, and this SNP-combination was detected in numerous other models and subpopulations, hence the interaction was most probably real.

No interactions could be detected for four of the SNPs in any of the models with co-variates (see Additional files 8 and 9). These were rs10103355 (ASAH1), rs2066509 (GCLC), rs10122075 (ACER2), and rs9306515 (CERKa), although the latter did reveal one interaction for diastolic blood pressure in the Supine model. However, in contrast to rs10103355 and rs2066509 the latter two SNPs only had one and two heterozygous genotypes in the entire population, respectively (see Additional file 2). All other subjects were homozygous for one of the genotypes. The number of interactions each SNPs participated in varied widely, the minimum being zero and the maximum 68% of all possible interactions. The maximum number of interactions in one or more of the subpopulations in all models for all SNPs varied but on average approximately half of possible interactions were detected and over 80% were detected in some subpopulations (see Additional files 8 and 9). I.e. the SNP-SNP interactions (and hence gene-gene interactions) were extensive and varied between models and between subpopulations within models.

The single SPTLC1 SNP (rs45461899) had 37 interactions (0.2% of all possible interaction) in the Supine and UL2 models (18 and 19, respectively) but none in the other models. In both models the interactions were only found in one subpopulation (14 and 10, respectively) and the number of interactions ranged from 8.9% to 20.0% of all possible interactions in these two subpopulations and hence the SNP do probably influence the blood pressure in these subpopulations.

In summary, the above observations suggest that models with low or no interactions in one or several subpopulations were less reliable. In addition, the lesser goodness-of-fit of models with small numbers of subjects in one or more subpopulations, which were particularly prominent in models with no covariates included (not shown), suggests that such models should be considered with caution.

### The Supine model

The distribution of cases in subpopulations and the number of interactions is summarized in Table 3 for the Supine model with and without covariates included in the LPA-SEM procedure (for a graphical view see Additional file 5). On average ~30% of all possible interactions were detected after correction for multiple testing in the model including covariates and a little less in the model without covariates included. There were no differences in the number of interactions between systolic and diastolic blood pressure, but there was a significant difference in the distribution of interactions between models with and without covariates included in the models.

The relative genetic variance in the subpopulations of the Supine model is summarized in Table 4. Almost 700 of the 990 (~70%) possible interactions were detected in one or more subpopulations. The interactions were on average detected in 6 subpopulations. A few differences were present for the two blood pressure traits: four interactions for the diastolic blood pressure were not found for the systolic blood pressure; and nine interactions for the systolic blood pressure were not found for the diastolic blood pressure (see Additional file 10). Interestingly, all of these were exclusively present in subpopulation 14, the hypertensive subpopulation *par excellence *(Table 4).

**Table 4:** Summary of epistasis and the hypertensive state in the Supine model

Subpopulation			1	2	3	4	5	6	7	8	9	10	11	12	13	14	Entire population
																	

Number of subjects			39	111	259	33	189	300	132	89	237	141	163	40	166	21	

																	

Genetics																	

																	

Diastolic blood pressures																	

Number of interactions			307	316	294	334	257	310	307	238	276	337	302	336	306	148	676

Percentage of possible (990)			31,0%	31,9%	29,7%	33,7%	26,0%	31,3%	31,0%	24,0%	27,9%	34,0%	30,5%	33,9%	30,9%	14,9%	71,5%

Number of interactions curated^3^			101	136	126	120	111	142	130	114	119	140	143	119	121	94	

Percentage of possible (253)			39,9%	53,8%	49,8%	47,4%	43,9%	56,1%	51,4%	45,1%	47,0%	55,3%	56,5%	47,0%	47,8%	37,2%	

Genetic values^b^:																	

Average			0,0338	0,0069	0,0032	0,0430	0,0049	0,0021	0,0059	0,0102	0,0031	0,0057	0,0039	0,0244	0,0049	0,1532	

Median			0,0260	0,0060	0,0020	0,0233	0,0032	0,0014	0,0040	0,0077	0,0022	0,0041	0,0032	0,0180	0,0037	0,1081	

Maximum			**0,1689**	0,0322	0,0191	**0,3166**	0,0285	0,0107	0,0371	0,0472	0,0182	0,0283	0,0214	**0,1359**	0,0259	**0,5209**	

Minimum			0,0022	0,0003	0,0000	0,0029	0,0000	0,0001	0,0002	0,0002	0,0001	0,0002	0,0001	0,0015	0,0001	0,0099	

																	

Systolic blood pressures																	

Number of interactions			307	318	298	230	257	321	306	238	275	334	295	336	307	174	681

Percentage of possible (990)			31,0%	32,1%	30,1%	23,2%	26,0%	32,4%	30,9%	24,0%	27,8%	33,7%	29,8%	33,9%	31,0%	17,6%	68,8%

Number of interactions curated^a^			102	136	128	104	111	144	130	114	120	141	143	119	122	103	

Percentage of possible (253)			40,3%	53,8%	50,6%	41,1%	43,9%	56,9%	51,4%	45,1%	47,4%	55,7%	56,5%	47,0%	48,2%	40,7%	

Genetic values:																	

Average			0,0334	0,0069	0,0032	0,0468	0,0049	0,0021	0,0060	0,0103	0,0031	0,0058	0,0040	0,0244	0,0048	0,1663	

Median			0,0257	0,0059	0,0020	0,0335	0,0032	0,0014	0,0040	0,0077	0,0023	0,0042	0,0033	0,0180	0,0035	0,1116	

Maximum			**0,1687**	0,0321	0,0192	**0,3208**	0,0285	0,0108	0,0372	0,0472	0,0183	0,0283	0,0214	**0,1360**	0,0260	**0,5209**	

Minimum			0,0021	0,0002	0,0000	0,0052	0,0001	0,0001	0,0002	0,0004	0,0001	0,0001	0,0001	0,0015	0,0002	0,0089	

																	

Blood pressures																	

																	

Diastolic blood pressures																	

	Mean		***60,0***	69,9	68,3	***71,5***	82,9	75,2	89,5	79,7	88,3	79,1	95,6	***86,2***	78,6	***112,6***	80,1

	Std. Deviation		***4,2***	4,1	3,5	***4,0***	2,1	2,2	6,9	1,5	3,3	2,8	5,0	***2,0***	6,1	***8,2***	10,1

	Std. Error		***0,7***	0,4	0,2	***0,8***	0,2	0,1	0,6	0,2	0,2	0,2	0,4	***0,3***	0,5	***1,8***	0,2

	Lower Bound (95% CI)		***58,6***	69,1	67,9	***69,8***	82,6	75,0	88,3	79,4	87,9	78,6	94,8	***85,5***	77,7	**108.9**	79,7

	Upper Bound (95% CI)		***61,4***	70,7	68,8	*73.2*	83,2	75,5	90,7	80,0	88,7	79,5	96,4	***86,8***	79,5	***116,3***	80,6

	Minimum		***49,5***	60,5	58,5	***63,0***	78,0	69,5	73,5	76,5	79,0	70,5	81,0	***82,0***	62,0	***98,0***	49,5

	Maximum		***69,0***	79,5	79,0	***79,5***	89,5	81,0	107,5	84,0	97,5	84,5	108,5	***91,0***	92,5	***132,5***	132,5

																	

Systolic blood pressures																	

	Mean		***97,8***	118,1	109,2	***117,0***	123,8	118,3	159,0	119,3	133,9	132,8	152,3	***139,4***	138,5	***182,5***	128,8

	Std. Deviation		***4,6***	6,1	5,3	***11,9***	4,7	5,5	13,3	5,0	6,1	6,9	10,4	***3,7***	8,9	***12,3***	17,8

	Std. Error		***0,7***	0,6	0,3	***2,1***	0,3	0,3	1,2	0,5	0,4	0,6	0,8	***0,6***	0,7	***2,7***	0,4

	Lower Bound (95% CI)		***96,3***	117,0	108,5	***112,8***	123,2	117,7	156,7	118,3	133,2	131,7	150,7	***138.3***	137,1	177,0	128,0

	Upper Bound (95% CI)		***99,3***	119,3	109,8	***121.2***	124,5	118,9	161,3	120,4	134,7	134,0	153,9	***140,6***	139,8	***188.1***	129,6

	Minimum		***84,5***	99,0	93,5	***96,51***	108,0	98,0	122,0	106,5	111,0	117,5	105.5	***132,5***	116,0	***158,5***	84,5

	Maximum		***105,5***	130,5	124,0	**139,5**	135,5	132,5	189,0	131,5	147,0	150,5	179,5	***148.0***	162,0	***211,0***	211,0

																	

	Hypertensive subjects (%)																

		Diastolic	***0***	0	0	***0***	0	0	47,7	0	16	16,3	88,3	***42,5***	42,2	***100***	

		Systolic	***0***	0	0	***0***	0	0	91,7	0	25,7	0	87,1	***2,5***	1,2	***100***	

																	

	Distribution of age groups																

		1	***82,1***	1,8	62,5	***21,2***	43,9	49,8	0,0	61,8	35,9	1,4	11,0	***2,5***	0,0	***19,0***	

		2	***17,9***	9,9	30,1	***18,2***	45,0	37,2	5,3	34,8	43,0	22,7	39,3	***20,0***	0,5	***47,6***	

		3	***0,0***	61,3	7,4	***42.4***	11,1	12,3	31,8	3,4	20,7	61,7	38,7	***57,5***	24,7	***33,3***	

		4	***0,0***	27,0	0,0	***18,2***	0,0	0,3	62,9	0,0	0,4	14,2	11,0	***20,0***	74,7	***0,0***	

																	

	Distribution of hypertensive subjects in age groups																

																	

	1	Diastolic	***0***	0	0	***0***	0	0	0	0	34,1	0	88,9	***0***	0	***100***	

		Systolic	***0***	0	0	***0***	0	0	0	0	18,8	50	83,2	***0***	0	***100***	

	2	Diastolic	***0***	0	0	***0***	0	0	57,1	0	32,4	0	96,9	***0***	0	***100***	

		Systolic	***0***	0	0	***0***	0	0	100	0	15,7	31,2	90,6	***62,5***	0	***100***	

	3	Diastolic	***0***	0	0	***0***	0	0	52,4	0	28,6	0	84,1	***0***	0	***100***	

		Systolic	***0***	0	0	***0***	0	0	85,7	0	26,5	13,8	93,7	***34,8***	58,5	***100***	

	4	Diastolic	***0***	0	0	***0***	0	0	48,2	0	0	0	66,7	***25***	1,6	***100***	

		Systolic	***0***	0	0	***0***	0	0	96,4	0	0	0	88,9	***50***	41,3	***100***	

^a ^Restricted to one interaction per gene-pair

^b ^Relative gene tic variance of phenotypic variance

The subpopulations in bold have relative genetic variance above 0.05.

Six SNP-interactions were detected in all the subpopulations for diastolic blood pressure (see Additional file 11). Three of these were also present in all the systolic blood pressure subpopulations, while the other three were detected in all the subpopulations less one. The interactions with the maximum values were exactly the same for the two blood pressure measurements and were almost the same in the subpopulations. These interactions identified some of the components of the rheostat, but details of the identified pathway were missing. For instance, sphingosine-1-kinase 1 (SPHK1) was not detected which would be the "missing link" between the ceramidases and the SP-1 lyase. Including all interactions detected in at least 13 of the 14 subpopulations captured SPHK1 and in particular placed the enzyme in the right position of the network (see Additional file 12). Some of the genes with more than one entrance differed in the influence of their SNPs depending on the subpopulation. However, none of the maximum genetic variances were captured by restricting the analysis to the most common interactions.

Unique interactions were detected in 5 of the 14 subpopulations amounting to almost 9% of all possible interactions (see Additional file 13). The maximal relative genetic variance was detected for the interaction between rs243887 (SPTLC3) and rs17159388 (CerS4) and amounts to 52% of the phenotypic variance in subpopulation 14. Two of the genes (SPTLC3 and CERKa) were only involved in interactions for diastolic blood pressure, and 4 genes (SPHK1, SPHKAP, SGPL1, and GLCM) were only detected for systolic blood pressure. However, genetic variations in these genes did not define any subpopulations unambiguously.

The number of interactions, the size of the relative genetic variance and distribution of hypertensive subjects in the subpopulations are summarised in Table 4. Four of these had relative genetic variance above 0.1 (subpopulations 1, 4, 12, and 14), while the remaining subpopulations had values below 0.05. No hypertensive subjects were detected in subpopulation 1 and 4, while all the subjects were hypertensive in subpopulation 14 regardless of age. In subpopulation 12 almost 50% of the subjects were hypertensive and they tend to be in the older age groups. These four subpopulations amounted to 6.9% of the entire populations and 10.1% of the diastolic hypertensive and 6.3% of the systolic hypertensive subjects.

Calculation of single SNP heritabilities i.e. relative additive + dominant variance [33] only revealed a total of 21 SNPs in 10 genes restricted to 5 subpopulations with heritabilities above 0.01 (see Additional file 14), that is only 0.14% of all possible main effects for single SNPs were detected. In subpopulation 14 exclusively consisting of hypertensive subjects nine SNPs had substantial heritabilities, though several of the genes in the kernel (in particular SPHK1) did not show any heritabilities above 0.01.

In the entire population 12.1% of the subjects had a diastolic blood pressure above 90 mmHg, 18.0% of the subjects had a systolic blood pressure above 140 mmHg, and 8.8% of the subjects had blood pressures above both thresholds. In a case-control design using these definitions of hypertension the maximal single-gene heritabilities did not exceed 0.0034 and were all represented by the same three SNPs in ACER1, ACER2, and CERKa. The numbers of significant epistasis were substantially lower than in the Supine model amounting to 55.25% of all possible epistasis. The maximal relative genetic variance (epistasis) was 0.0172, again substantially lower than in any subpopulation in the Supine model.

#### Networks

Sixteen of the 23 genes were connected in all subpopulations with a few exceptions, while the remaining 7 only connected to various degrees in all the subpopulations in the Supine model (see Additional files 15 and 16). Some of the latter genes signified differences of importance in some of the sub-pathways in the rheostat. This was most pronounced by the ceramide synthases, which differs in substrate specificity [43]. The rheostat network from subpopulation 14, the diastolic blood pressure, omitting all intragenic interactions (possible LDs), was the most simple and most comprehensive of all the subpopulation networks (see Additional file 17). The network contains 94 links (37.2% of all possible), but 3 genes were not connected at all (CerS1, CerS6, and ELOV3). A few more connections were present in the systolic network (not shown), but the same genes were captured (and missed). In particular, the rate-limiting enzyme SPTLC3 were connected in all networks, but the analogue SPTLC1 was only connected in subpopulation 14.

Subpopulations 1 and 14 represent the most non-hypertensive and the most hypertensive subpopulations, respectively. Although most links are present in both subpopulations networks it was clear that there were substantial and complex difference between the subpopulations of their connections in the rheostat network. Similar patterns were seen when comparisons between diastolic and systolic blood pressures were done within the subpopulations. A detailed interpretation of these differences is very complex, but generally the analysis showed that different SNPs, genes or subnetworks are of importance in discerning hypertensive from non-hypertensive, between different hypertensive subpopulations, and between diastolic and systolic blood pressures. Thus, the entire population consisted of at least 14 subpopulations that differed in importance of their components.

For diastolic blood pressure the centrality indices for ACER1 and SKHKAP were the most important in subpopulation 14 (see Additional file 15). Most remarkable was, however, GCLM which turned out to be the central node in many of the subpopulations. GCLM is a regulator of GCLC in the glutathion metabolism in response to oxidative stress [44], but is itself devoid of catalytic activity. GCLM was the central node in three of the four subpopulations with large genetic values (subpopulations 1, 4, and 12), the exception being subpopulation 14 in which ACER1 presented itself with highest centralities. In contrast, for systolic blood pressure generally the most important nodes were those in the kernel of sphingosine-1-phosphate metabolism (ASAH1, SPHK1, SPHKAP, and SGPP2). The subpopulations differed somewhat in importance of these genes, but short of a few additions (see Additional file 16), the regulation of the systolic blood pressure was linked to this subnetwork of the rheostat.

**Figure 4:**
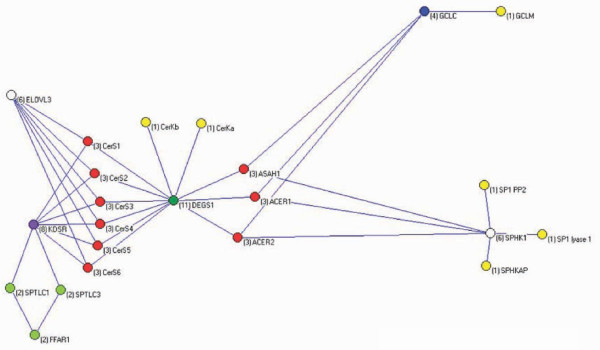
**The core biochemical network of the ceramide/sphingosine-1-phosphate rheostat**. A comprehensive core network is depicted including the possible source of free fatty acids synthesized *de novo *in the vascular bed (the fatty acid elongation enzyme, ELOV3), as well as the oxidative-redox system (GCLC and GCLM) which regulates neutral sphingomyelinase activity, a source of ceramides. These latter are only introduced as "summary" variables as the focus has been on the *de novo *pathway of ceramide synthesis. Node degrees are shown in the brackets.

**Figure 5:**
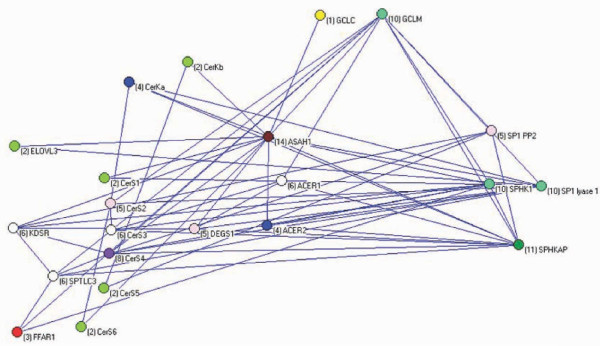
**The networks constructed for all the subpopulations in the Supine model are merged including the interactions with maximal genetic values producing the network shown**. This network compiles all detected interactions including the core metabolic network as shown in Figure 4 as well as indirect interactions.

**Figure 6:**
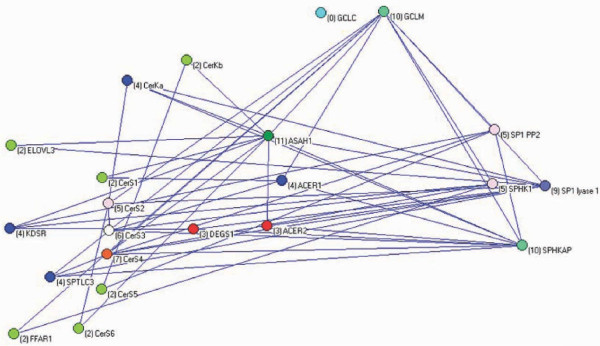
Subtracting the core network in Figure 4 from the complexed network in Figure 5 generate a network of epistasis excluding the metabolic flux.

The basic biochemical network of the rheostat is shown in Figure 4, a merger of all the networks of the subpopulations is shown in Figure 5, and the network remaining after extraction of the former from the latter is shown in Figure 6. Excluding pathway-direct regulatory intermediates in the sphingolipid metabolism the latter network heuristically could be interpreted as representing a superimposed regulatory network of the sphingolipid metabolism. The epistatic links of the superimposed regulatory network accounted for 83% of all the links in the Supine model.

## Discussion

This study establishs the ceramide/sphingosine-1-phosphate rheostat network to have substantial influence on blood pressure regulation. The approach presented identified the components of the rheostat network, but the exact definition of the core structure of the rheostat was hampered by a layer of interactions, which may be interpreted as regulatory structure keeping the rheostat (tightly) controlled. Nevertheless, it was possible to pinpoint central components of the composite network, regardless if the component are located in the core biochemical rheostat network *per se *or are parts of regulatory structures. The network is much more complex than indicated by the consensus biochemical pathways, for the simple reason that epistasis introduced as links in the network includes all kinds of interactions, not just the rather simple directed biochemical pathways (metabolic flux). It has been argued that correlation between nodes may decrease exponentially with distance and in large networks only nearest neighbour interactions are actually observable [45], but obviously this is not the case here. Regulatory structures encompass the level of feedback mechanisms in the biochemical pathways beyond direct metabolic fluxes including dynamic changes of cellular membranes caused by the composition of the sphingolipids in membranes [46], possible regulatory functions of metabolic intermediates, spatial organization and interaction of the components of the rheostat, as well as regulation of transcriptional activity and integration, all of which we have increasing but still fragmentary knowledge about. This complexity is shown in Figures 4-6. It should be noted that the networks shown were strictly curetted only including the maximal interactions between the nodes in each subpopulation. The number of interactions are much larger (almost 700) and non-redundant as different SNPs interact in different combinations with different relative epistatic variance in different subpopulations.

The results suggests that 1) the fatty acid components of the ceramides are not synthesized locally, but are derived from external sources (extensive involvement of FFAR1, tiny involvement of ELOV3); 2) only a restricted number of ceramide analogous are of general importance (CerS1 and CerS6 are not captured in most subpopulation); and 3) the regulatory structures differs between diastolic and systolic blood pressure, the former related to the oxidative stress status and the latter to the state of phosphorylation of sphingosine. It has recently been shown that several of the enzymes in the rheostat are influenced by the oxidative status of the cell including sphingomyelinases [47-49]. These are of course just summary statements and it is clear that all the subpopulations differ in importance of the components in the network and not the least their impact of the rheostat on blood pressure regulation. Finally, as no SNP exclusively defined any subpopulation this would suggest that the phenotypic variation is mediated by a network of genetic variations rather than a mutation in a single gene [32,50], i.e. the blood pressure regulation is truly polygenic. This statement is supported by the modest results of the many large genome wide studies of single-gene effects as mentioned in the Background and as demonstrated in this report.

This study has been focussed on the *de novo *pathway of ceramide synthesis, but the source of free fatty acid were touch upon by including genes representing import and *de novo *synthesis of free fatty acids, respectively. Also genes regulating the generation of ceramides from sphingomyelin were included, but were not evaluated in detail. Nevertheless, it turns out that the *de novo *pathway may be the most important source of ceramides considering the high relative genetic variance in the hypertensive subpopulation 14. But it is clear that supply of ceramide from hydrolysis of sphingomyelin is of significance, suggested by the strong involvement of GCLM-GCLC, which regulates (among other processes) the activity of the sphingomyelinases [51]. This has to be elucidated by extending the approach to include all candidate genes in the network.

The main interest in this study has been on the ceramide/sphingosine-1-phosphate network, but the genetic influence was only substantial in four of the subpopulations for the rheostat. Other networks (probably connected to the rheostat) are operating and it must be envisioned that many more subtypes of blood pressure regulation and hypertension are present beyond those identified in this study. Nevertheless, a genetic-metabolic network is identified as a target for intervention in up to 10% of individuals with essential hypertension, which, when confirmed and exact points of intervention are identified, represent a substantial increase in patients which potentially could benefit from personalized therapeutic intervention. In fact, the number of patients with a clear deviation from normal physiology would be tripled.

The approach presented here and that presented previously for diabetes mellitus type 2 [17] are the same, but differ in the selection of genes in the analysis. In the present presentation an established metabolic network was analysed, while in the diabetes study several seemingly unrelated genes associated with diabetes detected in numerous independent studies were included. In the latter study almost no main effect could however be detected in that particular population, but partitioning the population and analysis of variance revealed the importance of epistasis to the same degree as shown in the present study. This lends support to the notion that solitary detection of single-SNP associations with a condition may be of real significance although they mostly cannot be confirmed in different populations [52]. Both of the studies stress the importance of modelling physiological variables and in particular including general populations and avoiding dichotomizing variables (or rely on clinical endpoints) as they notoriously lose information [53]. As shown here and in the diabetes study [17] the use of continuous variables to partition of a random selected study population into several subpopulations provides far more information than is usually obtained by dichotomizing variables as done in a case-control study. This is true particular for polygenic conditions such as hypertension and diabetes, as everybody has a blood pressure and consume glucose only that some deviates from a healthy state in these processes. This probably also holds for most of the so-called Mendelian or monogenic conditions, because the penetrance is often not perfect, i.e. even such powerful "monogenes" depends on the state (genotype) of the genes in the network they are a part of.

## Conclusions

The approach presented in this study could potentially identify all the networks involved in the regulation of the blood pressure when extended to the entire genome. The latter is most desirable considering large fraction of interactions detected beyond the core biochemical network, which is merely a candidate gene approach with local interactions that will not capture essential regulatory structures or components such as transcription factors or miRNA. Genome-wide genotyping with suitably distributed SNPs should be very fruitful in the approach presented here. The approach should be efficient in detecting the network of genes of importance in any polygenic condition or trait provided that essential phenotypic variables are available.

## Authors' contributions

MF conceived, designed, encoded the R-scripts, performed the analysis, and drafted the manuscript. AL and TJ manages the Monica database and supplied basic phenotypic information. SM performed the insulin analysis and JEO performed the analysis of Supar. KS designed the analysis of the oxidative stress-related genes. JJ supplied the hypertension data. All authors read, commented and approved the manuscript.
